# The ALADINO Study: A National Study of Prevalence of Overweight and Obesity in Spanish Children in 2011

**DOI:** 10.1155/2013/163687

**Published:** 2013-09-08

**Authors:** Napoleón Pérez-Farinós, Ana M. López-Sobaler, M. Ángeles Dal Re, Carmen Villar, Estefanía Labrado, Teresa Robledo, Rosa M. Ortega

**Affiliations:** ^1^Spanish Agency for Food Safety and Nutrition, Ministry of Health, Social Services and Equality, C/ Alcalá 56, 28071 Madrid, Spain; ^2^Department of Nutrition, Faculty of Pharmacy, Complutense University of Madrid, 28040 Madrid, Spain

## Abstract

The aim of the study was to determine the prevalence of overweight and obesity in children in Spain using different sets of cut-off criteria, through a community-based cross-sectional study. The study was conducted in a representative sample of Spanish children between 6 and 9 years, recruited in Spanish schools, between October 2010 and May 2011. 7,569 boys and girls were selected. All were weighed and measured, and their parents were asked about their socioeconomic background, food habits and physical activity. The BMI of each was calculated, and the prevalence of overweight and obesity was determined by age and sex using Spanish reference tables (SPART), IOTF reference values, and WHO growth standards. The prevalence of overweight in boys ranged from 14.1% to 26.7%, and in girls from 13.8% to 25.7%, depending on the cut-off criteria. The prevalence of obesity in boys ranged from 11.0% to 20.9%, and in girls from 11.2% to 15.5%. The prevalence of obesity was the highest among those same children when using the SPART or WHO criteria. Overweight and obesity remain widespreading among Spanish children; a consensus on the definition of overweight and obesity cut-off criteria is necessary.

## 1. Introduction

Child obesity has become a serious public health problem [1] and has shown an alarming trend in growth in recent years across Europe [2], including Spain [3]. Overweight and obesity are associated with cardiovascular and cerebrovascular disease, diabetes, high blood pressure, dyslipidaemia, locomotor abnormalities, and even some forms of cancer—diseases with high rates of mortality and morbidity that place health systems under economic strain [2, 4]. Such morbidity can also lead to a loss of quality of life, sometimes early in life. Many children who are overweight before puberty remain overweight as young adults, bringing forward the mean age of onset of the above diseases [5, 6].

Tackling the child obesity epidemic requires accurate, up-to-date information be at hand. In Spain, the *Encuesta Nacional de Salud* (ENSE; the National Health Survey) provides data on overweight and obesity among children every three years [7]. However, the survey relies on self-declared height and body weight measurements, which reduces the accuracy of the final results. The last scientific study on child obesity in Spain that used objective measurements was the enKid [8] study—but that was back in 2000. These data are, therefore, now out of date. The measurement of overweight and obesity in children has the added difficulty that reference values for each age group and sex are required; unlike in adults, these conditions cannot be defined by single, fixed body mass index (BMI) values (i.e., overweight ≥25 and <30, and obesity ≥30 in adults). A number of criteria for defining overweight and obesity BMI in children exist, but the final prevalence values they supply can differ. Comparisons of obesity prevalence using different criteria have been done previously in other countries, and the differences between prevalences are significant [9–12]

The aim of the present work was to determine the prevalence of overweight and obesity in Spanish children aged 6–9 years, including regional differences, and to examine whether the different sets of cut-off criteria commonly used in such calculations lead to any important differences in the results obtained, within the ALADINO study. Deeper analysis concerning health habits and social, economical, and cultural factors will be analysed later.

## 2. Materials and Methods

The ALADINO (*ALimentación, Actividad Física, Desarrollo INfantil y Obesidad—Food, Physical Activity, Child development and Obesity*) study was a cross-sectional study of Spanish children of primary school age (6–9 years inclusive) performed under the auspices of the *Agencia Española de Seguridad Alimentaria y Nutrición* (AESAN; Spanish Agency for Food Safety and Nutrition) between October 2010 and May 2011.

### 2.1. Sample Size and Design

For the present work, the sample size required in order to estimate proportions with an accuracy of 2% (*d* = 0.02) and for *α* = 0.05 was determined. The expected proportion of overweight/obese children was expected to be around 20%. A design effect of 1.2 was applied to estimate the final sample size.

The sample was selected using a multistep method to ensure it was representative of Spanish boys and girls aged 6, 7, 8, and 9 years. The sample was first stratified by geopolitical region. Simple random sampling was used to select one province from each region, except for those regions with only one province and for the Spanish autonomous cities of Ceuta and Melilla on the North African Coast. Each selected province was further stratified by the size of its population centres (<50,000, 50,000–100,000, 100,000–500,000, and over 500,000 inhabitants). Schools were then selected within each population stratum, again by simple random sampling. The number of private and public schools in each province was taken into account so that the proportions would be adequately represented in the final sample. At each school the classes corresponding to the target age groups were selected, and all children in each were selected too.

### 2.2. Data Collection

This study formed a part of the Childhood Obesity Surveillance Initiative (COSI), promoted by the European Office of the WHO. Seventeen member states are involved in recording data for children aged 6–9 years, using a common methodology to allow comparisons between countries [13]. The ALADINO study involved the original COSI questionnaires, translated and adapted for the Spanish population. A total of three questionnaires were employed, one for the head teacher of each participating school, one for the parents of participating children, and one for the research personnel, which was completed during personal interviews with each participating child. Further analyses will be done with school and family questionnaires' data.

In the personal interview, each child's body weight and height were measured. Weight and height were determined using a digital electronic balance (Tanita UM-076 scale) (range 0.1–150 kg, precision 100 g) and a portable stadiometer (Tanita Tantoise) (range 0–207 cm, precision 1 mm), respectively. For these measurements, subjects were barefoot and wore only underwear. Subject BMI was calculated as weight (kg)/height^2^ (m^2^). All anthropometric measurements were made at the schools in the morning and following norms set out by the World Health Organization [14]. The measures were made by research personnel who were specifically trained.

### 2.3. Statistics Analysis

The mean BMIs for the boys and girls separately, and for both sexes together, within each age group, were determined. The prevalence of overweight and obesity by sex and age group was also determined using three different sets of cut-off criteria: (a) the definitions of overweight and obesity reflected by percentiles in the reference tables for the Spanish population (SPART criteria) [15]. These criteria were published by the Spanish Orbegozo Foundation and were based in longitudinal and cross-sectional studies performed among children from Bilbao (Spain). Overweight was defined between the 85th and 97th percentiles, and obesity was defined over the 97th percentile; (b) using IOTF reference values (overweight: cut-off points corresponding to an adult BMI ≥ 25 and <30 kg/m^2^; obesity: cut-off point corresponding to an adult BMI ≥ 30 kg/m^2^) [16]; and (c) using the WHO growth standards (overweight: BMI ≥ 1 SD and <2 SD; obesity: BMI ≥ 2 SD) [17]. The prevalence of overweight + obesity using the different criteria was also determined for both sexes together within each Autonomous Spanish Regions.

The McNemar test for independent samples was used (comparing two sets of data each time) to determine the significance of the differences in the prevalence values of overweight and obesity returned using these different sets of cut-off criteria. This test was performed for both sexes together and for boys and girls separately, for all age groups. The Student *t* test was used to compare the BMIs of the girls and boys within age groups. All calculations were performed using SPSS v.19 software.

## 3. Results

The required sample size was determined to be 1,844 children for each age group; the total required was therefore 7,376. The actual sample size was 7,659 (3,931 boys and 3,728 girls) from 144 schools. 22 schools (11.7%) rejected to participate in the study.

Table 1 shows the mean bodyweights, heights, and BMIs of the boys and girls in the different age groups. No significant difference was seen between the BMI of the girls and boys within any age group. 

The prevalence of overweight was found to be significantly higher when determined using the WHO standards and the IOTF values than when using the SPART criteria (*P* < 0.001 in all cases). No significant differences were seen between the percentage of overweight children as determined by the WHO standards and IOTF values, except between boys and girls aged 6, for whom the WHO standards gave a significantly higher value (Table 2). 

The prevalence values for obesity were significantly higher when calculated using the WHO standards than when determined using the other sets of criteria—except for (1) the girls in the 9 years age group, for which the SPART criteria returned the (significantly) highest value, and (2) for boys in the same age group, for which the WHO and SPART criteria returned similar results, both of which were significantly higher than that returned using the IOTF reference values. The SPART criteria returned values significantly higher than those obtained using the IOTF reference values, except for girls in the 6 year age group (Table 3).

**Figure 1:**
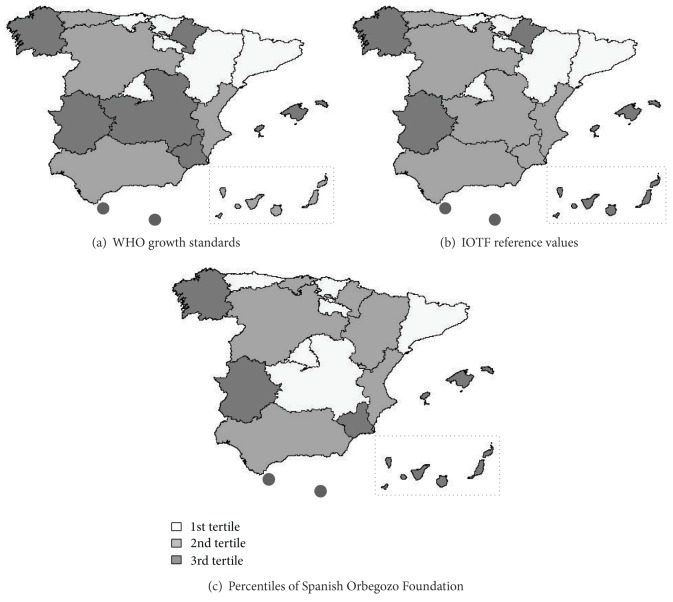
Prevalence of overweight and obesity in Spanish Autonomous Regions by BMI tertiles and three different criteria.

Figure 1 shows the joint prevalence for overweight + obesity for all children as a whole from each of the regions examined. Those of Catalonia, La Rioja, Madrid and the Basque Country fell into the first (lowest) tertile with all three sets of criteria, while those of the Balearic Islands, Extremadura and Galicia, and those from the cities of Ceuta and Melilla, fell into the third (highest) tertile, again with all three sets of criteria.

## 4. Discussion

Overweight and obesity in children are serious public health problems in Spain and other developed countries. Their prevalence in Spain has been increasing in recent years, as revealed by studies and surveys performed at both state and regional level [7, 18–22]. According to data published in 2006 by the ENSE, the prevalence of overweight in children aged 5–9 years was 21.4% (19.5% in boys and 23.4% in girls) [3]. These figures are lower than those recorded in the present study when prevalence was determined using the IOTF or WHO criteria, but higher than those recorded by the SPART criteria. The prevalence of obesity in 5–9 year-olds, according to the same ENSE data, was 15.4%, 15.1% in boys and 15.7% in girls (using IOTF criteria) [3]. These figures are lower than those recorded in the present study when using the SPART and WHO criteria, but higher than those determined using the IOTF reference values. It should be borne in mind, however, that the ENSE data are self-declared; they may therefore underestimate the true prevalence of child obesity [23].

According to the enKid study [8], the prevalence of overweight in 6–9 year-olds, as measured using the SPART criteria, was 16.0%, while in the present study it was 13.9% when measured in the same way. For girls, the prevalence of overweight was reported as 13.9% in the enKid study and was 13.8% in the present study. The prevalence of obesity in the enKid study, again measured using the SPART criteria, was 21.7%, while in present study it was 19.8% when using the same cut-off same criteria. In girls, the prevalence of obesity was 9.8% in the enKid study, and 14.5% in the present study. This was the only significant difference between the findings of these studies. With the exception of the latter difference, the present results suggest a stabilising of overweight and obesity in Spain, rather than the suspected increase [7, 18–22]; this agrees with that recorded in other countries [24, 25].

The prevalences of overweight and obesity recorded in the present study using the WHO standards or the IOTF reference values were higher than those recorded in France [26], England [27], and Portugal [28], lower than those recorded in Italy [29], and similar to those recorded in Malta [30]. The prevalence of overweight recorded in the present work using the WHO standards was also higher than that reported for the USA in girls and boys aged 6–11 years using the same criteria, although the prevalence of obesity among these American boys was greater than that recorded (using the same criteria) in the present work for Spanish boys [31].

In Spain, the NAOS (*Nutrición, Actividad Física y Prevención de la Obesidad*; Nutrition, Physical Activity and Prevention of Obesity) Strategy [32] of the Ministry of Social Services and Equality insists on the need to establish adequate information sources regarding nutrition and overweight/obesity and to create an “obesity observatory” in order to remain up to date in this area [33]. This has been endorsed by the *Ley de Seguridad Alimentaria y Nutrición* (Law on Food Safety and Nutrition) of 2011 [34]. The ENSE [7] collects data on the body weight and height of children from 2 years of age onwards, but it relies on self-declared data, and there is a strong possibility that any prevalence of obesity figures estimated from the calculated BMIs will be an underestimate. The fact that data are collected every three years has the advantage that trends can be established, but any individual values need to be used with caution. The enKid study [8], which was undertaken in 2000, was the last performed in Spain that involved objective data collection. The notable demographic changes that have occurred in the Spanish population in recent years therefore render the present study's results all the more important. 

Since the high mortality and morbidity figures associated with chronic disease were first made widely known, efforts have been stepped up to modify the factors that influence them, including obesity, diet, and physical activity [2, 35]. The results of the present work indicate that child obesity remains a major public health problem in Spain, and that the efforts that have been made over the years to stem it are no less required today. Prevention should still be understood as the main pillar in this battle; people therefore need to be made aware of healthy habits so that potentially harmful behaviours can be altered. A healthy, balanced diet and the need for regular exercise should be promoted at school, in the workplace, and at family, community, and health service level. Education remains the cornerstone in the fight against obesity, and strong cooperation between different parties is needed if the best results are to be obtained [33, 36].

The fact that obesity levels might have reached a plateau may mean that the resources put into fighting this epidemic are beginning to meet with success. However, it would be dangerous to think that the battle is won and that from now on obesity figures are likely to fall. Rather, efforts need to be increased to bring the prevention message to as many people as possible and should be accompanied by studies and the development of information sources that can help optimise the designation of resources.

The present work highlights the problem of using different criteria to define overweight and obesity in children. The prevalence of overweight was recorded as significantly lower when the SPART criteria were used rather than the IOTF reference values or WHO standards, with no significant differences between the results provided by these latter two sets of criteria. However, with respect to the prevalence of obesity, the results recorded by all three systems were significantly different to one another. Such a situation can only lead to confusion and a lack of confidence in the reliability of any results made known. To date, no consensus exists regarding which set of criteria should be used. Both the IOTF reference values and the WHO growth standards were produced with the idea of providing a common set of criteria that could be used with any population. However, their existence has led to even greater confusion, with parties often choosing the set of criteria that best matches their opinions or best suits their interests [37]. 

The main strengths of the present study lie in the sample size, and the sample's representativeness of the population of Spanish children aged 6–9 years (which took into account of different geopolitical regions, different sized population centres, and types of school). The methodology laid out in the COSI was also used. The results obtained are therefore reliable and render the ALADINO study the most authoritative with respect to the prevalence of child overweight and obesity in Spain. The main limitation of the study lies in the fact that at the regional level, while the representativeness of the sample is assured at the level of “all children aged 6–9,” it is less reliable for the different sexes and age groups taken separately (in particular for the smaller regions). For better results at this geopolitical level, studies using the same methodology but specific for each region would have to be performed.

## 5. Conclusions

The results show that overweight and obesity remain widespreading among Spanish children, although a plateau could have been reached. An East-West gradient in the overweight and obesity prevalence is observed in Spain. Periodic studies on overweight and obesity prevalence should be conducted to assess the trends. The overweight and obesity prevalences are significantly different when measured with different criteria. A consensus on the definition of overweight and obesity cut-off criteria is needed.

## Figures and Tables

**Table 1:** Weight, height, and body mass index by sex and age (mean and standard deviations).

	Weight (kg)		Height (cm)		BMI (kg/m^2^)	
	Mean	SD	Mean	SD	Mean	SD
Both sexes						
All	30.4	7.5	129.5	8.5	17.9	2.9
6 years	24.9	4.7	120.9	5.6	16.9	2.3
7 years	28.5	5.9	126.6	5.9	17.6	2.9
8 years	31.8	6.6	132.1	6.0	18.1	2.9
9 years	35.7	7.7	137.3	6.3	18.8	3.2
Boys						
All	30.7	7.5	129.9	8.4	18.0	2.9
6 years	25.1	4.7	121.4	5.5	17.0	2.2
7 years	28.9	6.0	127.1	5.6	17.8	3.0
8 years	31.9	6.5	132.4	6.0	18.1	2.7
9 years	36.1	7.6	137.7	6.2	18.9	3.2
Girls						
All	30.1	7.5	129.0	8.6	17.8	2.9
6 years	24.7	4.7	120.5	5.6	16.9	2.4
7 years	28.0	5.8	126.1	6.1	17.5	2.7
8 years	31.7	6.7	131.8	5.9	18.1	3.0
9 years	35.3	7.8	136.9	6.5	18.7	3.2

BMI: body mass index; SD: standard deviation.

**Table 2:** Prevalence of overweight, by sex and age, as determined using the different sets of criteria.

	*n*	Prevalence of overweight (95% CI)						McNemar test for comparison of proportions returned by the different sets of criteria		
		SPA*	95% CI	IOTF^†^	95% CI	WHO^‡^	95% CI	SPA-WHO^§^	IOTF-WHO^|^	SPA-IOTF^¶^
Both sexes								*P*	*P*	*P*
All	7,659	14.0	(13.2–14.8)	24.2	(23.2–25.2)	26.2	(25.2–27.2)	<0.001	<0.001	<0.001
6 years	1,829	11.1	(9.7–12.5)	19.4	(17.6–21.2)	24.5	(22.6–26.5)	<0.001	<0.001	<0.001
7 years	1,861	13.2	(11.6–14.7)	24.5	(22.5–26.4)	25.8	(23.8–27.7)	<0.001	0.183	<0.001
8 years	1,961	14.9	(13.4–16.5)	26.1	(24.1–28.0)	26.6	(24.6–28.5)	<0.001	0.629	<0.001
9 years	2,009	16.5	(14.9–18.1)	26.4	(24.5–28.3)	27.7	(25.7–29.6)	<0.001	0.219	<0.001
Boys										
All	3,931	14.1	(13.0–15.2)	23.8	(22.4–25.1)	26.7	(25.3–28.1)	<0.001	<0.001	<0.001
6 years	927	10.5	(8.5–12.5)	17.9	(15.5–20.4)	26.2	(23.4–29.1)	<0.001	<0.001	<0.001
7 years	954	12.1	(10.0–14.1)	23.5	(20.8–26.2)	25.9	(23.1–28.7)	<0.001	0.121	<0.001
8 years	1,018	15.8	(13.6–18.0)	26.3	(23.6–29.0)	26.5	(23.8–29.3)	<0.001	0.948	<0.001
9 years	1,032	17.7	(15.3–20.0)	26.8	(24.3–29.5)	27.9	(25.2–30.6)	<0.001	0.542	<0.001
Girls										
All	3,728	13.8	(12.7–15.0)	24.6	(23.3–26.0)	25.7	(24.3–27.1)	<0.001	0.051	<0.001
6 years	901	11.7	(9.6–13.8)	21.0	(18.3–23.6)	22.8	(20.1–25.5)	<0.001	0.046	<0.001
7 years	907	14.3	(12.0–16.6)	25.5	(22.7–28.3)	25.6	(23.1–28.3)	<0.001	1.000	<0.001
8 years	943	14.0	(11.8–16.2)	25.9	(23.1–28.7)	26.6	(23.8–29.4)	<0.001	0.569	<0.001
9 years	977	15.2	(13.0–17.5)	26.1	(23.3–28.8)	27.4	(24.6–30.2)	<0.001	0.219	<0.001

*Prevalence calculated using SPART criteria (BMI ≥ P_85_ and <P_97_).

^†^Prevalence calculated using IOTF reference values (cut-off points corresponding to an adult BMI ≥ 25 and <30 kg/m^2^).

^‡^Prevalence calculated with WHO growth standards (BMI ≥ 1 SD and <2 SD).

^§^McNemar test for comparison of prevalence of overweight returned using the SPART and WHO criteria.

^|^McNemar test for comparison of prevalence of overweight returned using IOTF and WHO criteria.

^¶^McNemar test for comparison of prevalence of overweight returned using the SPART and IOTF criteria.

**Table 3:** Prevalence of obesity, by sex and age, as determined using the different sets of criteria.

	*n*	Prevalence of obesity (95% CI)						McNemar test for comparison of proportions returned by the different sets of criteria		
		SPA*	95% CI	IOTF^†^	95% CI	WHO^‡^	95% CI	SPA-WHO^§^	IOTF-WHO^|^	SPA-IOTF^¶^
Both sexes								*P*	*P*	*P*
All	7,659	16.8	(16.0–17.6)	11.0	(10.3–11.7)	18.3	(17.4–19.2)	<0.001	<0.001	<0.001
6 years	1,829	11.5	(10.0–12.9)	10.4	(9.0–11.8)	15.0	(13.4–16.6)	<0.001	<0.001	0.016
7 years	1,861	15.8	(14.1–17.4)	12.4	(10.9–13.9)	19.1	(17.3–20.9)	<0.001	<0.001	<0.001
8 years	1,961	18.0	(16.3–19.7)	10.4	(9.1–11.8)	19.0	(17.3–20.7)	0.001	<0.001	<0.001
9 years	2,009	22.4	(20.3–24.5)	11.4	(9.8–13.0)	20.9	(18.8–23.0)	<0.001	<0.001	<0.001
Boys										
All	3,931	19.3	(18.0–20.5)	10.9	(9.9–11.8)	20.9	(19.6–22.2)	<0.001	<0.001	<0.001
6 years	927	14.0	(11.8–16.2)	9.9	(8.0–11.8)	16.3	(14.0–18.7)	<0.001	<0.001	<0.001
7 years	954	18.6	(16.2–21.1)	12.8	(10.7–14.9)	22.1	(19.5–24.8)	<0.001	<0.001	<0.001
8 years	1,018	20.2	(17.7–22.7)	10.1	(8.2–11.9)	21.5	(19.0–24.1)	0.001	<0.001	<0.001
9 years	1,032	23.6	(21.0–26.2)	10.7	(8.8–12.6)	23.2	(20.6–25.8)	0.344	<0.001	<0.001
Girls										
All	3,728	14.2	(13.1–15.3)	11.2	(10.2–12.2)	15.5	(14.4–16.7)	<0.001	<0.001	<0.001
6 years	901	8.8	(7.0–10.7)	10.9	(8.9–12.9)	13.6	(11.4–15.8)	<0.001	<0.001	<0.001
7 years	907	12.7	(10.6–14.9)	12.0	(9.9–14.2)	16.0	(13.6–18.4)	<0.001	<0.001	0.109
8 years	943	15.6	(13.2–17.9)	10.8	(8.8–12.8)	16.3	(13.9–18.6)	0.065	<0.001	<0.001
9 years	977	19.2	(16.7–21.7)	11.2	(9.2–13.1)	16.2	(13.9–18.5)	<0.001	<0.001	<0.001

*Prevalence calculated using SPART criteria (BMI ≥ P_97_).

^†^Prevalence calculated using IOTF reference values (cut-off point corresponding to an adult BMI ≥ 30 kg/m^2^).

^‡^Prevalence calculated with WHO growth standards (BMI ≥ 2 SD).

^§^McNemar test for comparison of prevalence of overweight returned using the SPART and WHO criteria.

^|^McNemar test for comparison of prevalence of overweight returned using IOTF and WHO criteria.

^¶^McNemar test for comparison of prevalence of overweight returned using the SPART and IOTF criteria.
